# Melanocortin*-1* receptor, skin cancer and phenotypic characteristics (M-SKIP) project: study design and methods for pooling results of genetic epidemiological studies

**DOI:** 10.1186/1471-2288-12-116

**Published:** 2012-08-03

**Authors:** Sara Raimondi, Sara Gandini, Maria Concetta Fargnoli, Vincenzo Bagnardi, Patrick Maisonneuve, Claudia Specchia, Rajiv Kumar, Eduardo Nagore, Jiali Han, Johan Hansson, Peter A Kanetsky, Paola Ghiorzo, Nelleke A Gruis, Terry Dwyer, Leigh Blizzard, Ricardo Fernandez-de-Misa, Wojciech Branicki, Tadeusz Debniak, Niels Morling, Maria Teresa Landi, Giuseppe Palmieri, Gloria Ribas, Alexander Stratigos, Lynn Cornelius, Tomonori Motokawa, Sumiko Anno, Per Helsing, Terence H Wong, Philippe Autier, José C García-Borrón, Julian Little, Julia Newton-Bishop, Francesco Sera, Fan Liu, Manfred Kayser, Tamar Nijsten

**Affiliations:** 1 Division of Epidemiology and Biostatistics, European Institute of Oncology, Via Ramusio 1, Milan, 20141, Italy; 2 Department of Occupational Health, University of Milan, Milan, Italy; 3 Department of Dermatology, University of L’Aquila, L’Aquila, Italy; 4 Department of Statistics, University of Milan Bicocca, Milan, Italy; 5 Department of Biomedical Sciences and Biotechnologies, University of Brescia, Brescia, Italy; 6 Division of Molecular Genetic Epidemiology, German Cancer Research Center, Heidelberg, Germany; 7 Department of Dermatology, Instituto Valenciano de Oncologia, Valencia, Spain; 8 Department of Dermatology, Brigham and Women’s Hospital and Harvard Medical School, Boston, MA, USA; 9 Channing Laboratory, Department of Medicine, Brigham and Women’s Hospital and Harvard Medical School, Boston, MA, USA; 10 Department of Epidemiology, Harvard School of Public Health, Boston, MA, USA; 11 Department of Oncology and Pathology, Cancer Center, Karolinska Institutet, Stockholm, Sweden; 12 Perelman School of Medicine at the University of Pennsylvania, Philadelphia, PA, USA; 13 Department of Internal Medicine and Medical Specialties, University of Genoa, Genoa, Italy; 14 Department of Dermatology, Leiden University Medical Center, Leiden, The Netherlands; 15 Murdoch Childrens Research Institute, Royal Children’s Hospital, Victoria, Australia; 16 Menzies Research Institute Tasmania, University of Tasmania, Hobart, Australia; 17 Servicio de Dermatologia, Hospital Universitario Nuestra Señora de Candelaria, Santa Cruz de Tenerife, Spain; 18 Institute of Forensic Research, Krakow, Polandfv; 19 Department of Genetic and Pathology, Pomeranian Medical University, Polabska, Poland; 20 Department of Forensic Medicine, University of Copenhagen, Copenhagen, Denmark; 21 Division of Cancer Epidemiology and Genetics, National Cancer Institute, NIH, Bethesda, MD, USA; 22 Unit of Cancer Genetics, Istituto di Chimica Biomolecolare, CNR, Sassari, Italy; 23 Dptd. Oncologia medica y hematologia, Fundacion Investigation Hospital Clinico Universitario de Valencia- INCLIVA, Valencia, Spain; 24 Department of Dermatology, University of Athens, Andreas Sygros Hospital, Athens, Greece; 25 Division of Dermatology, Washington University, St. Louis, MO, USA; 26 Dermatological R&D Skin Research Department, POLA Chemical Industries, Yokohama, Japan; 27 Shibaura Institute of Technology, Tokyo, Japan; 28 Department of Dermatology, Oslo University Hospital, Oslo, Norway; 29 Department of Dermatology, University of Edinburgh, Edinburgh, UK; 30 International Prevention Research Institute, Lyon, France; 31 Department of Biochemistry and Molecular Biology, University of Murcia, Murcia, Spain; 32 Department of Epidemiology and Community Medicine, University of Ottawa, Ottawa, Canada; 33 Section of Epidemiology and Biostatistics, Leeds Institute of Molecular Medicine, University of Leeds, Leeds, UK; 34 UCL Institute of Child Health, London, UK; 35 Department of Forensic Molecular Biology, Erasmus MC University Medical Center, Rotterdam, The Netherlands; 36 Department of Dermatology, Erasmus MC University Medical Center, Rotterdam, The Netherlands

**Keywords:** Genetic epidemiology, Melanoma, Meta-analysis, Pooled-analysis, Skin cancer, Study design

## Abstract

**Background:**

For complex diseases like cancer, pooled-analysis of individual data represents a powerful tool to investigate the joint contribution of genetic, phenotypic and environmental factors to the development of a disease. Pooled-analysis of epidemiological studies has many advantages over meta-analysis, and preliminary results may be obtained faster and with lower costs than with prospective consortia.

**Design and methods:**

Based on our experience with the study design of the Melanocortin-1 receptor (*MC1R*) gene, SKin cancer and Phenotypic characteristics (M-SKIP) project, we describe the most important steps in planning and conducting a pooled-analysis of genetic epidemiological studies. We then present the statistical analysis plan that we are going to apply, giving particular attention to methods of analysis recently proposed to account for between-study heterogeneity and to explore the joint contribution of genetic, phenotypic and environmental factors in the development of a disease. Within the M-SKIP project, data on 10,959 skin cancer cases and 14,785 controls from 31 international investigators were checked for quality and recoded for standardization. We first proposed to fit the aggregated data with random-effects logistic regression models. However, for the M-SKIP project, a two-stage analysis will be preferred to overcome the problem regarding the availability of different study covariates. The joint contribution of *MC1R* variants and phenotypic characteristics to skin cancer development will be studied via logic regression modeling.

**Discussion:**

Methodological guidelines to correctly design and conduct pooled-analyses are needed to facilitate application of such methods, thus providing a better summary of the actual findings on specific fields.

## Background

Since millions of Single Nucleotide Polymorphisms (SNPs) were identified by the SNP Consortium [1], a growing number of studies have reported the association of SNPs in candidate genes with several diseases. However individual studies of typical size usually have low statistical power to find true associations given the polygenic nature of most common diseases, leaving alone the various forms of potential interactions between genetic, phenotypic and environmental factors. The advent of genome-wide association studies allowed genotyping of hundreds of thousands of SNPs across the genome on a usually large number of subjects, but information on a wide spectrum of epidemiological and lifestyle factors were seldom collected, although the role of these factors in complex diseases is undoubtedly crucial.

Meta-analysis of genetic epidemiological studies has been adopted to increase the power of smaller candidate gene studies by summarizing results from multiple studies. However the lack of access to individual data precludes in-depth investigations, including analyses of gene-gene and gene-environment interaction, and appropriate stratified analyses. This may potentially lead to false-positive or false-negative results, or biased magnitudes of associations, as previously pointed out [2].

Pooled-analysis of the primary data has been shown to have critical methodological advantages over meta-analysis [3,4] and has been applied successfully in the genetic epidemiology field [4-11]. Pooled-analysis uses standardized definitions of cases, outcomes and covariates, as well as the same analytical methods, thus limiting potential sources of heterogeneity across different studies. It also allows investigators to better control for confounding factors, evaluate alternative genetic models and estimate the joint effect of multiple genes. Finally, population-specific effect and gene-gene and gene-environment interactions may be better assessed using pooled-analysis [12]. The pooling of data from observational studies has become more common recently, and different approaches of data analysis have been applied [13]. Methodological guidelines to correctly design and conduct pooled-analyses are needed to facilitate application of such methods, thus providing a better summary of the actual findings on specific fields. Moreover, the awareness of the potential problems connected with the establishment of international collaborations and data pooling might help investigators to avoid or overcome them.

We describe here our experience with the study design of an international pooled-analysis on Melanocortin-1 receptor gene, SKin cancer and Phenotypic characteristics (M-SKIP project). In the first part of the paper, we explain the procedures that were used to identify studies and to collect and standardize data. In the second part we describe the statistical analysis plan that we are going to apply, giving particular attention to methods of analysis recently proposed to account for between-study heterogeneity and to explore the joint contribution of genetic, phenotypic and environmental factors in the development of a disease.

## The M-SKIP project: rationale and aims

Melanocortin-1-receptor (*MC1R*, MIM#155555) is one of the major genes that determine skin pigmentation and it has been reported to be associated with risk of melanoma [14], possibly through the determination of the tanning response of skin to UV radiation [15-17]. However the relationship between some *MC1R* variants and melanoma also in darkly-pigmented European populations suggests that *MC1R* signaling may have an additional role in skin carcinogenesis beyond the UV-filtering differences between dark and fair skin [18]. In previous meta-analyses [14,19,20] authors found evidence of a significant association between melanoma, red hair and fair skin and the five *MC1R* variants R151C, R160W, D294H, D84E and R142H, and suggested a possible role in melanoma development, via non-pigmentary pathways, for I155T and R163Q variants. However, the specific contribution of each *MC1R* variant to melanoma development via pigmentary and non-pigmentary pathways could not be evaluated in meta-analyses due to the lack of individually joint information on *MC1R* variants and phenotypic characteristics.

The aim of the M-SKIP project is therefore to perform a pooled-analysis of individual data on sporadic skin cancer cases and controls with information on *MC1R* variants, in order to: 1) assess the association of *MC1R* variants with melanoma, basal cell carcinoma (BCC) and squamous cell carcinoma (SCC); 2) assess the association between *MC1R* variants and phenotypic characteristics, including hair and eye color, skin color, skin type, common and atypical nevi, freckles, and solar lentigines; and 3) perform stratified analyses on *MC1R* variants and skin cancer by phenotypic characteristics, and evaluate *MC1R*-phenotype interaction in skin cancer risk.

## Data collection and creation of the standardized dataset

### The identification of data sets and data collection

Published epidemiological studies on *MC1R* variants, melanoma, non-melanoma skin cancer (NMSC) and phenotypic characteristics associated with melanoma [21,22] were searched until April 2010 in the following databases: PubMed, ISI Web of Science (Science Citation Index Expanded) and Embase, using the keywords “MC1R” and “melanocortin 1 receptor” alone and in combination with the terms “melanoma”, “basalioma”, “basal cell carcinoma”, “squamous cell carcinoma”, “skin cancer”, “hair color”, “skin color”, “skin type”, “eye color”, “nevi”, “freckles”, and “solar lentigines”, with no search restriction. The computer search was supplemented by consulting the bibliographies of the articles and reviews. We also tried to identify unpublished datasets by personal communication with participant investigators, members of the Advisory Committee, and with attendees of scientific meetings. Unpublished datasets were evaluated by an internal peer-review process before inclusion.

We selected papers according to the following inclusion criteria: 1) observational studies on single-primary sporadic skin cancer cases with information on any *MC1R* variant or 2) control series with information on any *MC1R* variant and at least one phenotypic characteristic under study. Permanent exclusion criteria were: 1) populations selected for *MC1R* status or for other genetic factors, 2) studies including only familial and/or multiple-primary melanoma cases, because we wanted to study *MC1R*-melanoma association at a population level, therefore excluding cases for whom the role of genetics is probably stronger. In the first step of the project, we also excluded genome-wide association studies (GWAS), because their different study design and genotyping methodology would significantly increase the heterogeneity of our data; however GWAS with epidemiological data would be included in a next step of the project and their results would be compared with those of classical genetic epidemiological studies.

The original search provided 748 papers, among them 111 were considered potentially interesting and full-text articles were retrieved and evaluated. We excluded 49 articles for the following reasons: duplicate populations (N = 20), no data on outcome (case/control status or any of the studied phenotypic characteristics) or on *MC1R* variants (N = 12), case reports, commentaries or reviews (N = 6), GWAS (N = 6), populations selected for genetic factors (N = 4) and multiple primary melanoma cases only (N = 1). The remaining 62 independent studies were considered eligible for inclusion in the pooled-analysis.

For each independent study, we identified the corresponding investigator and retrieved his/her contact information. Each investigator was invited to join the M-SKIP project: this required them to sign a participation form and a document attesting to approval of the study guidelines, and then to provide their data in electronic form without restrictions on format. A detailed list of variables relevant for skin cancer was provided and, for each available variable in the list, the authors were required to compile a form with a clear and complete description on how it was collected and coded. Investigators did not send any personal identifier with data, but only identification codes. Finally, investigators were asked to send a signed statement declaring that the original study was approved by an Ethic Committee and/or that study subjects provided a written consent to participate in the original study.

Data collection started in May 2009 and was closed in December 2010. During this period, 43 investigators were contacted and invited to share data. Thirty-one (72%) agreed to participate and provided data on 28,998 subjects, including 13,511 skin cancer cases (10,182 melanomas) and 15,477 controls from 37 independent published [19,23-62] and 2 unpublished studies. Both the unpublished datasets came from investigators who were originally contacted for their published data and who had further data of (still) unpublished studies. Among the 12 non-participant investigators, seven did not reply to our invitation letter, three were not able to retrieve the original dataset and two were not interested in the project. The total number of skin cancer cases and controls from the 25 independent studies [63-95] of non-participant investigators was 5,135 and 8,262, respectively. The study design was case–control for 13 studies, control-only for 11 studies, and case-only for one study.

### Quality control, data coding and creation of the standardized dataset

We inspected the data for completeness and resolved inconsistencies with the investigator of each study. A number of subjects were excluded due to the following reasons: multiple-primary melanoma cases (N = 1596), missing data on *MC1R* variants (N = 1081), non-skin melanoma cases (N = 150), subjects with atypical mole syndrome and no skin cancer (N = 58), non first-primary melanoma cases (N = 24), familial melanoma cases, defined as subjects with two first-degree relatives or three or more any-degree relatives with melanoma (N = 25), other reasons including: unknown case/control status, duplicate subjects, or inappropriate controls (N = 232).

The following study-related variables were recoded uniformly: study country, study design, source of controls, application of case–control matching, methods to define phenotypic characteristics, genotyping methodology, whether genotyping was done in the same center for cases and controls and was blinded for case/control status, and DNA source. These variables were not used to assign a quality score to each study, but will be taken into account in meta-regression and sensitivity analyses. In addition, the variables listed in Table 1 were retrieved from each study if available, checked for quality, recoded in a standardized manner and entered in the main database. Finally, data on *MC1R* variants were entered for each subject. Quality controls and data coding were performed independently by two investigators, and inconsistencies were solved via consensus.

**Table 1:** List of the main variables, number of original studies and related subjects per variable

**Variable**	**Studies (%) N = 39**	**Melanoma cases (%) n = 7806**	**NMSC cases (%) n = 3151**	**Controls (%) n = 14875**
Age	37 (95%)	7761 (99%)	3150 (100%)	14550 (98%)
Gender	39 (100%)	7801 (100%)	3151 (100%)	14853 (100%)
Ethnicity	38 (97%)	6770 (87%)	3142 (100%)	13833 (93%)
Body mass index	8 (21%)	557 (7%)	1380 (44%)	2226 (15%)
Smoking status	6 (15%)	2266 (29%)	419 (13%)	2286 (15%)
Intermittent sun exposure	21 (54%)	4493 (58%)	1266 (40%)	2286 (15%)
Continuous sun exposure	21 (54%)	4909 (62%)	741 (24%)	1938 (13%)
Sunburns	25 (64%)	4210 (54%)	1288 (41%)	2968 (20%)
Artificial UV exposure	16 (41%)	3842 (49%)	298 (9%)	1058 (7%)
Family history of skin cancer	27 (69%)	6660 (85%)	1289 (41%)	3318 (22%)
Family history of cancer other than skin	19 (49%)	4445 (57%)	371 (12%)	1630 (11%)
Melanoma body site	24 (62%)	6271 (80%)	NA	NA
Melanoma histology	19 (49%)	4868 (62%)	NA	NA
Breslow thickness	24 (62%)	5907 (76%)	NA	NA
Hair color	34 (87%)	6841 (88%)	2590 (82%)	11889 (80%)
Eye color	31 (79%)	5990 (77%)	2456 (78%)	10720 (72%)
Skin color	23 (59%)	3517 (45%)	826 (26%)	2963 (20%)
Skin type	31 (79%)	6590 (84%)	1992 (63%)	4540 (31%)
Common nevi	19 (49%)	3817 (49%)	442 (14%)	1181 (8%)
Atypical nevi	11 (28%)	2681 (34%)	642 (20%)	1447 (10%)
Freckles	21 (54%)	4028 (52%)	737 (23%)	2333 (16%)
Solar lentigines	6 (15%)	1419 (18%)	442 (14%)	1088 (7%)

*NA* = not applicable; *NMSC* = non melanoma skin cancer.

Some variables were collected in different ways in different studies. We report here as an example the rules we used to standardize sun exposure variables, in order to provide suggestions on how to recode variables with highly heterogeneous assessment among studies.

Intermittent and continuous sun exposure was coded as hours of exposure per day if the information was structured in this way. If not, we converted it to hours/day on a scale of 0 as no exposure and 6 as the maximum hours of exposure per day. For example, for datasets with four classes of exposure (never, seldom, often, always), we recoded the classes as 0, 2, 4, 6 hours/day. If individual sun exposure was collected over different time periods, we calculated the average exposure weighting for years of exposure in each time period. Other continuous variables (i.e. days of exposure per year, average hours of exposure per year) were converted to hours/day using the following algorithm:

1) calculate the variable mean on all the study subjects as:

(1) $$\mu =\sum_{i=1}^{n}x_{i}/n$$

where *x*_*i*_ is the measure of the continuous variable on subject *i*, and *n* is the study sample size;

2) calculate the average hours of exposure/day (*ν*) over all the datasets with the variable coded (or recoded) in this way as in 1);

3) recode each observation basing on the proportion $x_{i}:\mu ={\hat{x}}_{i}:\nu$ as:

(2) $${\hat{x}}_{i}=\nu x_{i}/\mu$$

4) set as 6 (maximum hours of exposure per day) the value of all calculated values greater than 6.

The assumption underlying this coding was that the average sun exposure pattern for study subjects was similar for different studies (and countries). Since we will use this variable only for confounding adjustment and/or effect modifier analyses, the purpose was to regroup subjects with a similar pattern of sun exposure, although the precise individual amount of sun exposure could not be estimated.

As a general rule, when a variable (i.e. common nevi count) was collected into classes, we recoded each class by using its median. The maximum numbers for open categories were chosen according to the available M-SKIP data.

## Brief description of the collected data and statistical power

The final dataset was created in June 2011 and included data on 7,806 melanoma cases, 3,151 NMSC cases (2,211 BCC, 788 SCC and 152 with both), and 14,875 controls.

Distribution of data according to study country in which the study was performed is presented in Table 2. The majority of data came from Europe, especially from southern European populations. There was no significant difference in participation rate according to study area (Fisher exact test p-value: 0.25).

**Table 2:** Summary of data included in the M-SKIP project by geographical location

**Study area**	**Invited investigators**	**Participant investigators (studies)**	**Melanoma cases**	**NMSC cases**	**Controls**
Africa	1	0 (0)	0	0	0
Asia	3	2 (2)	0	0	345
Australia	4	2 (3)	744	298	290
Northern Europe^a^	8	6 (6)	858	1629	8095
Central Europe^b^	6	3 (4)	977	639	2398
Southern Europe^c^	9	8 (12)	2,747	0	2263
North America	13	11 (12)	2,480	585	1484
**TOTAL**	**43**^**d**^	**31**^**d**^**(39)**	**7808**	**3151**	**14875**

*NMSC* = non melanoma skin cancer.

^a^ includes Denmark, Norway, Sweden, The Netherlands, UK.

^b^ includes France, Germany, Poland.

^c^ includes Greece, Italy, Spain.

^d^ one investigator collected data for two different areas (North America and Asia).

The main characteristics of the studies included in the M-SKIP database are described in Table 3. The majority are case–control studies (54%) with population or healthy controls and case–control matching. Phenotypic characteristics were frequently assessed by self-administered questionnaire (41%) or examination by a dermatologist or research nurse (36%). The majority of studies sequenced the entire coding region of the *MC1R* (67%) and used blood as DNA source (62%).

**Table 3:** Main characteristics of the included studies

	**Studies (%) N = 39**	**Melanoma cases (%) n = 7806**	**NMSC cases (%) n = 3151**	**Controls (%) n = 14875**
**Study design**				
Case–control	21 (54%)	5092 (65%)	2052 (65%)	6852 (46%)
Case only	11 (28%)	2646 (34%)	0	0
Control only	6 (15%)	0	0	1464 (10%)
Cohort	1 (3%)	68 (1%)	1099 (35%)	6559 (44%)
**Source of controls**				
Hospital	6 (21%)	509 (10%)	1169 (37%)	1847 (12%)
Population or healthy^a^	21 (75%)	4651 (90%)	1982 (63%)	12872 (87%)
Mixed	1 (4%)	0	0	156 (1%)
**Case–control matching**^**b**^				
No	10 (45%)	3151 (61%)	1739 (55%)	9578 (71%)
Yes	12 (55%)	2009 (39%)	1412 (45%)	3833 (29%)
**Phenotype assessment**				
Self-administered questionnaire	16 (41%)	2768 (35%)	672 (21%)	1875 (13%)
Examination by an expert	14 (36%)	3970 (51%)	1380 (44%)	4392 (30%)
Instrumental measure	2 (5%)	0	0	222 (1%)
Mixed	5 (13%)	297 (4%)	1099 (35%)	7247 (49%)
No measure	2 (5%)	771 (10%)	0	1139 (8%)
**Genotype assessment**				
Sequencing analysis	26 (67%)	5942 (76%)	1059 (34%)	4813 (32%)
Others^c^	13 (33%)	1864 (24%)	2092 (66%)	10062 (68%)
**DNA source**				
Blood	24 (62%)	4645 (60%)	2743 (87%)	13304 (89%)
Buccal cells	14 (36%)	3161 (40%)	408 (13%)	1326 (9%)
Tissue	1 (3%)	0	0	245 (2%)

*NMSC* = non melanoma skin cancer.

^a^ healthy subjects are blood donors, friends or relatives of cases.

^b^ individual or frequency.

^c^ includes RFLP, SNaPshot, allele discrimination assay.

We calculated that the minimum required sample size to find a statistically significant association between a *MC1R* variant and melanoma assuming a similar association to that observed in our previous meta-analysis [14] (Odds Ratio (OR) = 1.5) is around 7,500 cases and 7,500 controls for rare variants (1-2% allele frequency in controls), and 1,400 cases and 1,400 controls for common variants (8-10% allele frequency in controls), with 90% statistical power. Sample size for gene-environment interaction analysis was also calculated with the program POWER, version 3.0 [96]. Considering the study of a simple two-way interaction between an environmental factor and a rare *MC1R* variant, around 5,000 cases and 5,000 controls would be needed to observe a multiplicative interaction effect of 2.0, arising to 16,000 cases and 16,000 controls to observe a smaller multiplicative effect of 1.5, both with 90% statistical power. For common *MC1R* variants, the same gene-environment interaction effects of 2.0 and 1.5 could be observed with around 1,200 cases and 1,200 controls, and with around 3,500 cases and 3,500 controls, respectively. Our sample size therefore is appropriate for the purpose of the analysis, and large enough to allow stratified and interaction analyses, especially to find even small interaction effects with the most frequent variants, and larger interaction effects for less common variants.

## Statistical analysis plan

### Appropriateness and representativeness of the collected data

Comparability of the main study population characteristics and results between studies included and excluded from the pooled-analysis will be assessed. Funnel plots to evaluate participation bias will be drawn and Egger’s test [97] will be performed.

Departure of genotype frequencies of each *MC1R* variant from expectation under Hardy-Weinberg equilibrium will be assessed by Chi Square test among controls for each study, in order to detect any possible genotyping error or stratification problem in the datasets.

### Combining data into a single dataset with random effects models

A first analysis using data combined into one dataset, is to fit them with logistic regression models with random slope. Considering a dominant model, let *π*_*ik*_*/X* be the probability of skin cancer for subject *i* (*i = 1,…,n*_*k*_) of study *k* (*k = 1,…,K*) conditional on the presence of a certain *MC1R* variant (*X*). We will account for the fixed *MC1R* effect and the random selection of studies, assuming a model that relates *MC1R* and study effects linearly to the logit of the probabilities:

(3) $$\text{logit}(\pi_{\mathrm{ik}}\text{/ }X)=\alpha +\beta X_{\mathrm{ik}}+b_{k}X_{\mathrm{ik}}$$

In this model the transformed regression coefficient exp(*β*) is the odds of skin cancer for a subject with the *MC1R* variant compared with a subject without the *MC1R* variant, and the *b*_k_ are the study-specific coefficients accounting for the random selection of studies, with *b*_k_ ~ N(0, *σ*^*2*^_*b*_), where *σ*^*2*^_*b*_ represents the between study variance of the *MC1R* effect.

The logistic regression model above described could be applied to different inheritance models and could include covariates, in order to adjust the studied associations by possible confounding factors. In order to include the available information from all the studies, missing values could be estimated in the model with multiple imputation and/or the creation of a missing-data indicator variable. However, when the majority of missing data are the results of non-availability of certain variables in some studies, as for the M-SKIP project, the use of both multiple imputation and the missing-data indicator would be likely to introduce a bias in comparison with the complete case method [98,99] and a two-stage approach would be preferred.

### Two-stage analysis with random effects models

The two-stage analysis method [100] will allow us to overcome the problem of the availability of different study covariates. The pooled-estimates of the association of *MC1R* variants with each skin cancer type and each phenotypic characteristic will be calculated as follows.

First, study-specific ORs will be calculated by applying logistic regression to the data from each study to estimate the odds of skin cancer conditional on the presence of a *MC1R* variant (*X*), controlling for confounders *Z*_*k*_. For study *k* (*k = 1,…,K*), assuming just one confounder, the model is written as:

(4) $$\text{logit}(\pi_{\mathrm{ik}}/X)=\alpha_{k}+\beta_{k}X_{\mathrm{ik}}+\gamma_{k}Z_{\mathrm{ik}}$$

where *π*_*ik*_ is the conditional probability of skin cancer for subject *i* (*i = 1,…,n*_*k*_) of study *k*. Although *MC1R* variants were uniformly defined across studies, the confounders *Z*_*k*_ may be specific to a particular study. Analyses with original covariates and with recoded data will be performed and compared. The exposure log-odds ratio for study *k* is denoted *β*_*k*_, the confounding log-odds ratio is denoted *γ*_*k*_, and the *α*_*k*_ are the study-specific intercepts. The *β*_*k*_ are assumed to vary across studies according to the second-stage model:

(5) $$\beta_{k}=\beta +b_{k}+e_{k}$$

where *β* is the pooled-exposure log-odds ratio, *b*_*k*_ are random effects with *b*_k_ ~ N(0, *σ*^*2*^_*b*_), where *σ*^*2*^_*b*_ represents the variability of the study-specific exposure effects *β*_*k*_ about the population mean *β,* and *e*_*k*_ are independent errors with *e*_k_ ~ N(0, *σ*^*2*^_*k*_), where *σ*^*2*^_*k*_ describes the within-study variation of the *β*_*k*_. In the first stage ${\hat{\beta }}_{k}$and its variance ${\hat{\sigma }}_{k}^{2}$ are estimated from equation 4, separately for each study.

The two-stage estimator of the pooled exposure effect *β* is a weighted average of the ${\hat{\beta }}_{k}$, weighted by the inverse marginal variances of the ${\hat{\beta }}_{k}$, denoted $w_{k}={\left({\hat{\sigma }}_{k}^{2}+\sigma_{b}{}^{2}\right)}^{-1}$. Thus:

(6) $$\hat{\beta }=(\sum_{k}w_{k}{\hat{\beta }}_{k})/\sum_{k}w_{k}$$

(7) $$\mathrm{var}(\hat{\beta })={\left(\sum_{k}w_{k}\right)}^{-1}$$

Two methods [100] are frequently used to estimate the random effects variance *σ*^*2*^_*b*_ in equations 6 and 7. These methods are pseudo-maximum likelihood and moment estimation.

### Investigation of heterogeneity among studies

Homogeneity among the study estimates will be measured by Q statistic and I-Square [101], the latter representing the percentage of total variation across studies that is attributable to heterogeneity rather than to chance. Meta-regression analysis will be performed to investigate heterogeneity among study estimates, by evaluating the role of methodological characteristics of the studies and the characteristics of study populations.

### Joint association of MC1R and phenotypic characteristics with skin cancer risk

Stratified analysis for the association of *MC1R* variants with each skin cancer type will be performed for different phenotypic characteristics. The hypothesis of homogeneity of ORs among strata will be verified using the Breslow-Day test [102].

**Figure 1:**
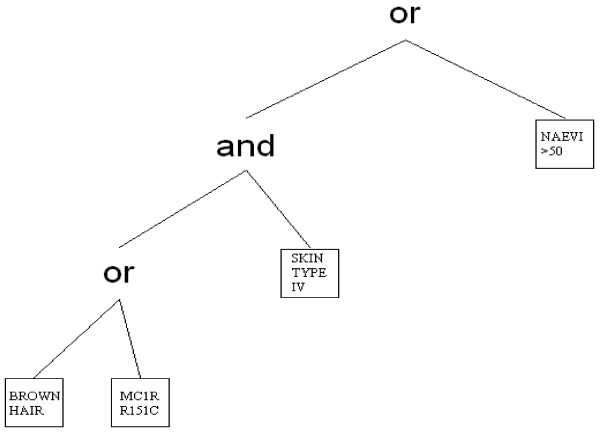
**Example of a logic tree representing the Boolean expression “*more than 50 common naevi*V [*skin type IV*Λ (*MC1R R151C*V*brown hair*)]”.**

In order to identify combinations of *MC1R* variants and phenotypic characteristics associated with each skin cancer type, we will perform logic regression, a recently proposed tree-based statistical method intended for binary predictors [103]. This approach is particularly useful for detecting subpopulations at high or low risk of disease, characterized by high-order interactions among covariates, and thus the methodology could be well applied to the study of complex diseases like cancer. First, we will dichotomize continuous and categorical variables by choosing appropriate thresholds and by creation of dummy variables. For phenotypic characteristics we will define dummy variables in order to 1) have as much differentiation as possible in hair and eye color, and 2) separate the extreme classes of skin type and common nevi count from intermediate classes, because it has been suggested [21] that in the meta- and pooled-analysis setting misclassification affects the intermediate classes of exposure more than the extreme ones. Let *X*_*1*_*, X*_*2*_*,…, X*_*p*_ be the binary predictors obtained by dichotomization, and let *Y* be the binary response variable (case/control status). The appropriate logic regression model can be written as:

(8) $$\text{logit}(Y=1|X_{1},X_{2},\ldots ,X_{n})=\alpha +\sum_{j=1}^{k}\beta_{j}L_{j}$$

where *L*_*j*_ is a Boolean expression of the predictors *X*_*i*_, such as $L_{j}=X_{4}^{C}\wedge (X_{5}\vee X_{1}\vee X_{3}^{C})$ with ∧=AND, ∨=OR and ${}^{C}=\text{NOT}$. Logic regression could be generally applied to any type of regression outcome as long as the proper scoring function is specified. For the logic regression model in equation 8, the goal is to find the Boolean expressions *L*_*j*_ that minimize the binomial deviance, with the parameters *β*_*j*_ and the Boolean expressions *L*_*j*_ estimated simultaneously. The output from logic regression is represented as a series of trees, one for each Boolean predictor *L*_*j*_, and the associated regression coefficient. An example of a logic tree that may be applied to our pooled analysis is shown in Figure 1. Using this representation it is possible to start from a logic tree and obtain any other logic tree by a finite number of operations such as growing of branches, pruning of branches, changing of predictors and/or operators.

The searching for the optimum combinations of *MC1R* variants and phenotypic characteristics mostly associated with skin cancer will be undertaken by a (stochastic) simulated annealing algorithm [104-106]. This algorithm has a good chance to find a model that has the best or close to best possible score but, in the presence of noise in the data, typically overfits data. In order to select the best model, application of a combination of cross-validation and randomization tests has been suggested [104,105].

In an explanatory setting, at risk gene-phenotype combinations will be identified among a very large number of possible combinations by logic regression-based methods recently proposed [106,107]. The skin cancer risk of the identified subpopulations will be estimated within the pooled-analysis context using the two-stage analysis previously described.

Structural equation models will be also applied to eventually clarify the independent and dependent role of *MC1R* variants on skin cancer by phenotypic characteristics.

Finally, the role of environmental exposure will be investigated by entering new covariates in the models, by subgroup analyses and by studying gene-environment and phenotype-environment interactions using traditional and new proposed methodologies [108].

### Use of MC1R data

In all the proposed analyses, each of the nine most frequently investigated *MC1R* variants (V60L, D84E, V92M, R142H, R151C, I155T, R160W, R163Q, D294H), as well as known rare mutations affecting *MC1R* function [109] will be evaluated assuming different inheritance models and choosing the one that fits the data best. Haplotype frequencies will be estimated using the iterative Expectation-Maximization algorithm [110,111], and their association with each skin cancer type and phenotypic characteristics will be evaluated. Moreover, for the studies that sequenced the entire gene, we will evaluate the impact on skin cancer and phenotypic characteristics of the total number of *MC1R* variant alleles and of the scores obtained from appropriate classification of *MC1R* variants [112].

## Discussion

Based on our experience with the study design of the M-SKIP project, we have described here the most important steps in planning, conducting and analyzing pooled individual data from genetic epidemiological studies. A previously published commentary highlighted the advantages and limitations of this kind of analyses, but did not describe the statistical methods that could be used to pool datasets [4]. Some methods for pooling results of epidemiological studies were suggested [10,100,112-114], but specific problems related to genetic epidemiology – such as the evaluation of different genotyping methodology, the Hardy-Weinberg equilibrium testing, the hereditary model assumption, and the assessment of gene-phenotype and gene-environment interaction – were not discussed.

Within the M-SKIP project, we collected a large amount of data in which multiple hypotheses can be examined with greater statistical power than is possible in individual studies. The response rate of invited investigators was high (72%), probably due to the well defined criteria of data collection and use, the clear publication policy, and the presence of an Advisory Committee tasked with monitoring adherence to project guidelines and scientific quality. Another strength of the pooled-analysis here described is the carefully-planned approach to standardizing the demographic, epidemiological and phenotypic information obtained from individual studies, giving the opportunity to perform appropriate and detailed subgroup and interaction analyses. Because the inclusion of an individual study in a particular analysis is not dependent on whether those investigators have published findings on that association, and because of inclusion of unpublished datasets, our pooled-analysis should not be affected by publication bias, as it might a meta-analysis of the published literature. Finally, we plan to analyze data by conventional and recently proposed statistical methods, and will compare and integrate the results obtained with these different approaches.

The main limitation of a pooled analysis, especially with respect to prospective consortia, is that it was planned retrospectively, and hence there was no *a priori* standardization of data collection. On the other hand, pooled-analysis may be feasible with fewer funds than those required for a prospective consortium, and it takes shorter time to obtain results because the original data have already been collected. The quality of genotype methodology may be heterogeneous among different participant laboratories. We will take into account this possible problem both by calculation of Hardy-Weinberg equilibrium and by meta-regression analysis. Finally, while we will try to assess the existence of participation bias, we cannot completely rule out that the results could be affected by the exclusions of the studies from the investigators who refused to participate in this pooled analysis.

In conclusion, the data collected within the M-SKIP project are a valuable resource for investigating associations between *MC1R* variants and skin cancer, particularly for population subgroups, and may be an appropriate setting to better investigate the genetics of sporadic skin cancer. A pooled-analysis of epidemiological studies is feasible, has many advantages over meta-analysis in making it possible to adjust for confounders and assess interactions, and in addition preliminary results may be obtained with lower costs and shorter time than with prospective consortia. We are convinced that its success depends upon the initial definition and approval of clear guidelines necessary for conducting such studies. The diffusion of pooled-analysis in genetic epidemiology field will assist epidemiologists and other health professionals in synthesizing the vast amount of available data on specific gene-disease associations and a common data-base would be the source of possible future investigations.

## Competing interests

The authors declare that they have no competing interests.

## Authors’ contributions

SR designed the study and directed its implementation, including quality assurance and control, wrote the paper and was the project leader. SG contributed to the design and supervision of the study and advised on recoding epidemiological data. MCF advised on recoding genetic data, is a member of the Advisory Committee of the project and contributed data to the study. VB, CS helped to carry out the statistical analysis plan of the study. PM contributed to the design and supervision of the study. RK, EN, JH, PG, NAG, TD, LB, RFdM, WB, TD, NM, GP, GR, AS, LC, TM, SA, PH, THW, FL, MK, TN and the GEM Study Group contributed data to the study. JH, PAK, MTL are members of the Advisory Committee of the project and contributed data to the study. PA, GCGB, JL, JNB, FS are members of the Advisory Committee of the project. All the members of the Advisory Committee were involved in the design and supervision of the study. All authors have revised and approved the manuscript.

## Pre-publication history

The pre-publication history for this paper can be accessed here:

http://www.biomedcentral.com/1471-2288/12/116/prepub
